# Evading Antivirus Detection Using Fountain Code-Based Techniques for Executing Shellcodes

**DOI:** 10.3390/s25020460

**Published:** 2025-01-15

**Authors:** Gang-Cheng Huang, Ko-Chin Chang, Tai-Hung Lai

**Affiliations:** 1 Department of Computer Science and Information Engineering, Chung Cheng Institute of Technology, National Defense University, Taoyuan 335009, Taiwan; qoo24571864@gmail.com; 2 Department of Electrical and Electronic Engineering, Chung Cheng Institute of Technology, National Defense University, Taoyuan 335009, Taiwan; kcchang@ccit.ndu.edu.tw

**Keywords:** antivirus evasion, fountain code, Metasploit framework, msfvenom, meterpreter, shellcode

## Abstract

In this study, we propose a method for successfully evading antivirus detection by encoding malicious shellcode with fountain codes. The Meterpreter framework for Microsoft Windows 32-bit and 64-bit architectures was used to produce the shellcode used in this investigation. The experimental results proved that detection rates were substantially decreased. Specifically, the number of detected instances using antivirus vendors for 32-bit shellcode decreased from 18 to 3, while for 64-bit shellcode, it decreased from 16 to 1. This method breaks up a malicious payload into many packets, each with their own distinct structure, and then encodes them. This obfuscation approach maintains the shellcode’s integrity, ensuring correct code execution. However, in the persistence phase of the penetration testing process, this method offers an additional means of evading antivirus techniques.

## 1. Introduction

Global internet users interact with software, retrieve information from search engines, and engage in entertainment due to the internet’s swift proliferation. Cybercriminals attack unsecured systems via the internet, jeopardizing user privacy and data integrity. Despite the prevalent utilization of antivirus software, innovative malware methodologies frequently circumvent detection, rendering systems susceptible to criminal access. Organizations often use endpoint detection software in conjunction with additional security measures to safeguard assets. Nonetheless, the deployment of hardware and software may be inadequate for preventing and identifying all destructive tactics employed by hackers. An increasing number of security specialists now perform penetration testing to identify systems’ vulnerabilities. During this process, specialists may exploit weaknesses to establish backdoors and maintain access [1]. However, they will face increasing technological challenges in doing so due to the continuous innovation of antivirus software technologies. Attack and defense are in conflict with one other in this respect [2].

Reconnaissance, weaponization, delivery, exploitation, installation, command and control (C2), and actions form the framework often known as the Cyber Kill Chain [3]. This model accounts for the assault life-cycle and the moments at which defense techniques may be executed successfully. Both attackers and cybersecurity experts can gather open-source intelligence, or OSINT, to identify possible risks and vulnerabilities [4]. Although cybersecurity tools can detect multiple payloads from the popular Exploit-DB [5], an issue-free system cannot be guaranteed [6]. The Cyber Kill Chain’s installation phase is the subject of the current study [7]. To improve shellcode obfuscation and lower antivirus detection rates, this study presents a method for encoding shellcodes using fountain codes.

This study emphasizes the application of fountain codes to evade antivirus measures for penetration testing and red team evaluations. Although these approaches may illustrate the constraints of existing security solutions, they also present considerable ethical dilemmas. The possible abuse of these tools for nefarious ends requires their rigorous regulation and supervision. Researchers and practitioners must guarantee these technologies’ responsible use, emphasizing the enhancement of cybersecurity defenses rather than facilitating exploitation.

The structure of this study is as follows: Section 2 reviews current advancements in shellcode obfuscation techniques and fountain code applications. Section 3 details the proposed methodology, focusing on the encoding and decoding processes using fountain codes. Section 4 presents the experimental setup and results, including shellcode generation, memory allocation, detection rates, and entropy analysis. Section 5 analyzes the limitations, decoding process, and security implications of the findings. Finally, Section 6 summarizes the contributions and proposes potential directions for future research.

## 2. Related Studies

### 2.1. Evasion and Detection Technique

Mechanisms for antivirus detection employ signatures, behavioral detection, heuristic detection, and sandboxing to recognize malware [8]. Antivirus evasion mechanisms used by hackers involve techniques such as inserting meaningless code and modifying signatures, obfuscating payloads, and encrypting malicious code using XOR. Additionally, they utilize polymorphisms with complex methods to complicate analysis, as well as process injection, through DLL injections or shellcode [9]. Current antivirus evasion methods include obfuscation, polymorphism, and encryption [10]. Obfuscation modifies the appearance of code without altering its functionality [11], while polymorphism ensures that each payload instance differs in structure [12].

Geng et al. (2024) thoroughly surveyed malware evasion techniques, classifying them into three categories: transformation-based, concealment-based, and attack-based strategies. The authors proposed a strategy-driven framework that integrated these techniques from the viewpoint of malware authors, illustrating how attackers employ various methods to circumvent detection. The study identified trends in evasion techniques, emphasizing the challenges faced by defenders and proposing future research directions to improve malware detection capabilities [13].

The shikata_ga_nai and powershell_base64 tools from msfvenom’s encoder are widely recognized for their ability to evade signature-based antivirus systems through polymorphic XOR encryption [14]. Similarly, Veil-Evasion generates diverse payloads in multiple languages (e.g., Python, C, and PowerShell) using advanced obfuscation techniques. Other methods, such as converting malicious code into ROP devices to inject PE headers [15,16], and tools like TheFatRat, combining encryption with anti-debugging strategies, further highlight the evolution of antivirus evasion techniques [17].

Control flow manipulation techniques, such as thread-hiding, suspending-threads, multi-threading, and self-debugging, bypass anti-debugging mechanisms by concealing debuggers and altering malware execution [18]. Past research on payload attack vectors for Windows and Linux highlights simplified malware injection techniques, such as ShellSwap, which utilizes jmp instructions for shellcode hopping, achieving an 88% success rate in evading detection [19].

Another study proposed the use of Multifaceted Deep Generative Adversarial Networks (MDGANs) to detect mobile malware, leveraging grayscale images from Android APKs, and API call sequences processed through GoogleNet and LSTM. The GAN-generated data enhanced the training set, achieving a 96.2% classification accuracy, surpassing the accuracy rates of existing malware detection frameworks [20]. While the proposed method leverages GANs to enhance training data, it heavily relies on existing malware datasets.

### 2.2. Fountain Code

Fountain codes, such as Luby Transform (LT) codes and Raptor codes, are engineered for efficient data transfer [21]. LT codes employ probabilistic methods to produce random data segments, facilitating effective decoding with a small number of packets. Raptor codes enhance LT codes by incorporating a preprocessing phase, which increases decoding efficiency and minimizes the necessary packet count, rendering both techniques appropriate for high-loss situations [22,23]. Raptor codes augment LT codes via preprocessing, thus improving decoding success rates and enhancing transmission efficiency [24]. These techniques proficiently address data loss and enhance performance in transmission systems, especially in contexts with unstable networks.

These are essential in network communication, especially in contexts with significant packet loss, such as wireless networks and peer-to-peer systems [25]. Their capacity to produce an unlimited number of encoded packets makes them exceptionally effective in unstable contexts, such as multicast and broadcast networks [26]. Decoding may commence upon receiving a sufficient quantity of packets, thus minimizing latency and enhancing data recovery efficacy. These codes are particularly efficient for real-time multimedia streaming, facilitating seamless video playing over shaky connections via incremental decoding [27]. Moreover, they are extensively utilized in cloud storage and content distribution networks (CDNs), as they guarantee dependable transmission and enhance storage management [28]. Fountain codes are, thus, crucial in contemporary network systems, as they reduce packet loss and retransmissions [29].

## 3. Methodology

### 3.1. Procedure

Algorithm 1 depicts the encoding process utilizing fountain codes. To augment data unpredictability and randomness, the procedure began by encoding the original shellcode while incorporating redundancies. Then, the Fisher–Yates shuffle was utilized to randomize the order of the created packets [30]. This randomization guaranteed that the data could be divided into an unlimited number of encoded packets, ensuring strong obfuscation against detection.
**Algorithm 1:** Shellcode encoding and validation using fountain code.**Data**: Original Shellcode Data**Result**: Validated and High-Entropy Encoded Shellcode
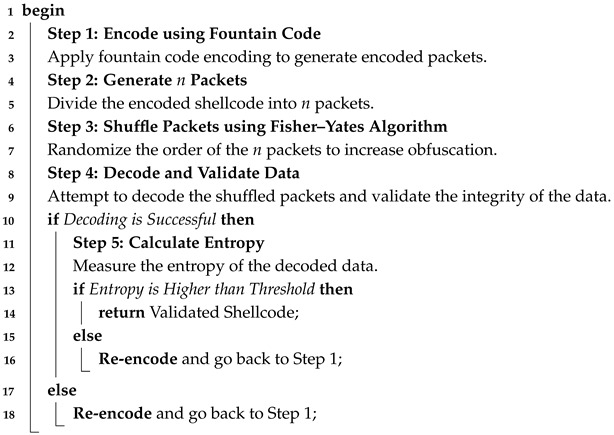


### 3.2. Encode and Decode

Fountain codes improve data transmission efficiency by generating encoded packets through random slice selection and XOR operations. Our method streamlined the encoding process by randomly selecting integers from within a range based on the number of slices (*X*). This approach balanced simplicity and performance, ensuring obfuscation and effective antivirus evasion, regardless of possible data loss.

Algorithm 2 illustrates the encoding process of the simplified fountain code. The encoding procedure produced *x* packets, each denoted by (I,C), where *I* represents the set of chosen slice indices and *C* represents the encoded data.

Algorithm 3 shows that the encoder produces *n* packets (I,C), with each packet represented as $p_{i}$ and the whole collection of encoded data represented by $P=\{p_{0},p_{1},\ldots ,p_{n}\}$. The decoded indices $I^{\prime }$ aggregates the indices from each accurately decoded slice, and the decoder operates by processing up to *x* slices. The encoding and decoding techniques guarantee that the reconstructed shellcode $P^{\prime }$ corresponds to the original shellcode *P*, thus maintaining data integrity.

The time complexity of the Simplified Fountain Code Encoder Algorithm (SFCEA) was primarily determined via the encryption step, which processed one packet of the encoded shellcode, resulting in a complexity of O(n). The algorithm then selected *d* unique indices from *X* slices and performed XOR operations on each slice, yielding a total complexity of O(X+d×l), where *l* represents the slice size.

For the Simplified Fountain Code Decoder Algorithm (SFCDA), the determination of the time complexity involved processing each packet through XOR operations and decryption. This resulted in a complexity of O(x×l+n). Both algorithms were scaled to reflect the size of the data being processed.

Although more advanced methods such as the Robust Soliton Distribution or preprocessing in Raptor codes can enable optimization, this simplified approach maintained a hierarchical structure, resulting in an overall time complexity of $O(n^{2})$. This design choice prioritized simplicity and comprehensibility, making it suitable for shellcode production, albeit with higher computational overhead.
**Algorithm 2:** Simplified Fountain Code Encoder (SFCEA)**Input**: *P*: byte array of shellcode,*l*: slice size (in bytes),*s*: encryption seed**Output**: Encoded packet (I,C) where*I*: slice indices;*C*: encoded data1**Step 1: Encrypt the shellcode:**2$P_{a}\leftarrow P_{a}⊕s,\;\forall a\in \left\{0,1,\ldots ,|P|-1\right\}$;3**Step 2: Calculate the number of slices:**4X←⌈|P|/l⌉;5**Step 3: Randomly select the degree:**6d∈[1,X];7**Step 4: Initialize the packet data:**8$C\leftarrow {\left\{0\right\}}^{l}$;9**Step 5: Randomly choose *d* indices:**10I⊆{0,1,…,X−1};
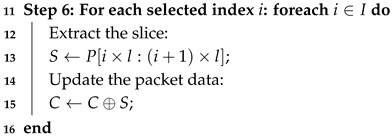
17**Step 7: Return the encoded packet: **(I,C);
**Algorithm 3:** Simplified Fountain Code Decoder Algorithm (SFCDA)**Input**: $C=\{c_{0},c_{1},\cdots ,c_{x}\}$: received encoded packets;*l*: size of each slice;*s*: encryption seed;*t*: total size of the shellcode**Output**: $P^{\prime }$: Byte array for the decoded plaintext shellcode1**Step 1: Initialization**2Initialize slices array and decoded indices set;3**Step 2: Process each packet**
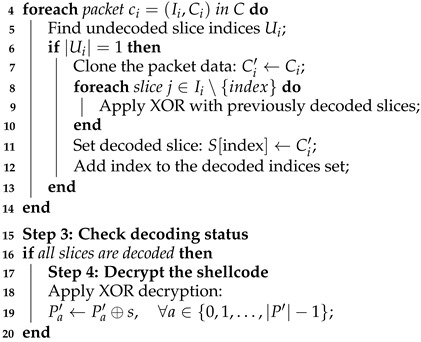
21**Step 5: Return result**22Return the decoded plaintext shellcode $P^{\prime }$;

### 3.3. Evaluating Data Randomness

To determine data randomness using shellcode and encoded data, scholars often use entropy [31,32]. The entropy method used in this study for determining the shellcode was previously used in [33].

To measure randomness, the normalized Shannon entropy $H_{\mathrm{norm}}$ was used to evaluate the unpredictability of encoded data:

$$H=-\sum_{b\in D}p\left(b\right)\cdot \log_{2}\left(p\left(b\right)\right),\;H_{\mathrm{norm}}=\frac{H}{H_{\max}}$$

where

$$H_{\max}=\log_{2}\left(|\{b|b\in D\}|\right).$$

Higher entropy indicates greater obfuscation and complexity, which is essential for avoiding detection.

## 4. Experiment and Results

### 4.1. Shellcode Generation and Execution

For our experiments, we used msfvenom, a payload generation tool from the Metasploit framework, to create the shellcode. This tool is commonly employed in penetration testing and research, enabling the customization of payload type, architecture, and output format [34]. The generated shellcode was executed using C# within the .NET Framework 4.7.2 [35].

The shellcode execution workflow used followed a series of steps, as detailed in Appendix A.3:

1. Memory Allocation: The VirtualAlloc function reserves memory with
  PAGE_EXECUTE_READWRITE permissions to prepare an execution environment for the shellcode;
2. Shellcode Loading: Marshal.Copy transfers the shellcode into the allocated memory;
3. Thread Creation: A new thread is created using CreateThread, with its entry point set to the shellcode memory address;
4. Thread Synchronization: The WaitForSingleObject function ensures that the thread completes execution before the process terminates.

This process ensured secure and efficient shellcode execution while maintaining precise control over memory and thread management.

### 4.2. Encoding Successes and Failures Compared

This experiment created shellcode using the msfvenom command, as shown in Table 1. The LHOST argument represents the attack’s IP address.

Each input parameter produced 25 samples. As seen in Table 2, Table 3, Table 4 and Table 5, short slices and insufficient packets prevented proper decoding. These results show that greater slices resulted in closer entropy between encoded and original data. An attacker could remotely control a target machine using a reverse shell. By connecting across a port on the victim’s device, the attack bypassed firewalls [36]. The entropy results presented summarize the impacts of varying packet counts and slice sizes on the encoding and decoding success rates, as well as on the entropy of the encoded data. Key observations include the following:

- **Redundancy and Packet Count:** Increasing packet counts (M= 500, 750, 1000, 2000, 3000, 4000) enhanced redundancy, improving decoding success rates. However, the improvement diminished as *M* exceeded 2N, indicating that redundancy beyond this threshold provided limited additional benefits.
- **Impact of Slice Size:** Smaller slice sizes (l=4,8) led to consistent decoding failures due to higher fragmentation and insufficient redundancy. Conversely, larger slice sizes (l=16,32,64,128) achieved 100% decoding success, demonstrating their ability to balance redundancy and fragmentation effectively.
- **Entropy Trends:** Larger slice sizes increased the encoded entropy (**Encode Entropy (MIN)** and **Encode Entropy (MAX)**), achieving values closer to those of the original shellcode entropy. For example, with l=64, **Encode Entropy (MAX)** remained consistently high (≈0.965) across varying packet counts, indicating effective data mixing.
- **File Size Growth:** Both packet count and slice size contributed to larger file sizes. For instance, increasing *M* from 1000 to 4000 nearly quadrupled the file size. This trade-off reflected the balance between ensuring decoding success and maintaining reasonable file sizes.

Table 2 and Table 3 present the lower entropy results for x86 and x64 shellcode, while Table 4 and Table 5 provide the higher entropy results. These results demonstrate the correlation between slice size and entropy, decoding success rates, and file size. The observations include the following:

1. Smaller slice sizes (l=4,8) consistently fail to decode due to insufficient redundancy;
2. Higher slice sizes (l=64,128) achieve optimal entropy while maintaining a balance between file size and decoding reliability.

These findings underscore the importance of parameter selection in optimizing shellcode obfuscation and detection evasion while preserving practical usability.

### 4.3. Scan Results for Malware

#### 4.3.1. Well-Known Evasion Tools

To compare our approaches with the evasion tactics presented in Appendix A.1, we utilized widely recognized encoding and encryption tools. In such experiments, the direct use of the original msfvenom executable backdoor during penetration testing on systems with antivirus software is inadvisable, as it has been identified by many antivirus vendors. Moreover, multiple antivirus engines have exhibited significant decreases in detection when employing shikata_ga_nai with 10 and 20 encoding iterations. While this method effectively circumvents certain signature-based detection systems, numerous antivirus programs still categorize the encoded payload as hazardous. The 32-bit malware payload was developed in C# and designed for execution via the Veil-Evasion program (version 3.1.14). We created 64-bit malware for further comparison with PwnWind, one of TheFatRat’s evasion modules.

#### 4.3.2. Proposed Method

All samples were subjected to testing on Kleenscan.com, a professional malware scanner that incorporates over 30 distinct antivirus vendors and does not disclose files during malware assessment [37]. Based on the data shown in Table 6, we concluded that the shellcode from the original file was generated via Meterpreter sourced from many vendors. Our method successfully encoded the shellcode, decreasing it from 18 to 3 and from 16 to 1. All of these findings were evaluated in October 2024.

Table 7 and Table 8 compare the detections between the original and encoded shellcode. This experiment demonstrated that, similar to the shellcode, the detection description was altered, reducing the detection rate. In the table, the antivirus providers are organized alphabetically according to the initial letter of each name. The 32-bit shellcode comprises 354 bytes with parameters defined by a seed number of 0x20, 16 slices, and 1000 packets, whereas the 64-bit shellcode consists of 510 bytes with the same parameters for seed number, slices, and packets.

Using this approach, it was possible to build encoded shellcode in a unique manner. As shown in Table 9, we used this method to generate malware that cannot scan all samples, which was ideal. As the sample that contained parameters generated via 32-bit shellcode in 354 bytes had a seed number of 0x00, 16 slices, and 250 packets, this sample was identified as dangerous using 12 different antivirus engines. Furthermore, according to Table 10, the sample in question was a 64-bit shellcode contained inside 510 bytes. It had a seed number of 0x00, 16 slices, and 2000 packets. It was identified as dangerous by five different antivirus engines. However, despite the fact that the encoded virus removed the characteristics from Msfvenom, it was necessary to combine additional tools and approaches to enhance the evasion strategy.

## 5. Discussion

### 5.1. Worst-Case Scenario Analysis

The dnSpy tool was utilized to decompile binaries created by the .NET Framework and .NET Core, enabling a deeper understanding of the encoding process [38]. To analyze the worst-case scenario, we used this tool to deconstruct the encoded shellcode and compare it with its original form.

**Figure 1:**
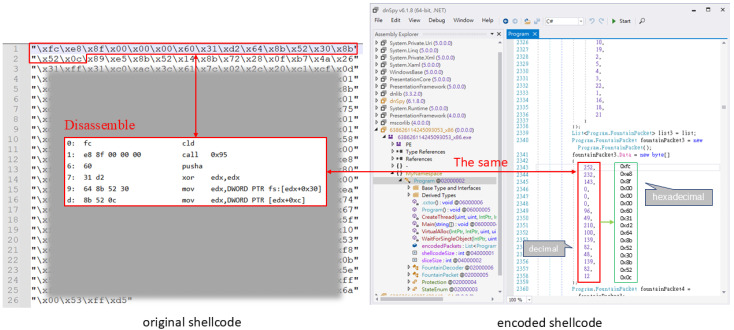
A comparison of the 32-bit shellcode before and after encoding in the worst-case scenario.

Figure 1 illustrates the comparison of the original and encoded 32-bit shellcode. We observe that the initial 14 bytes of both the original and encoded shellcode are identical (in decimal format). This lack of encryption represents a significant flaw in the encoding process, as it does not obfuscate critical shellcode features effectively.

**Figure 2:**
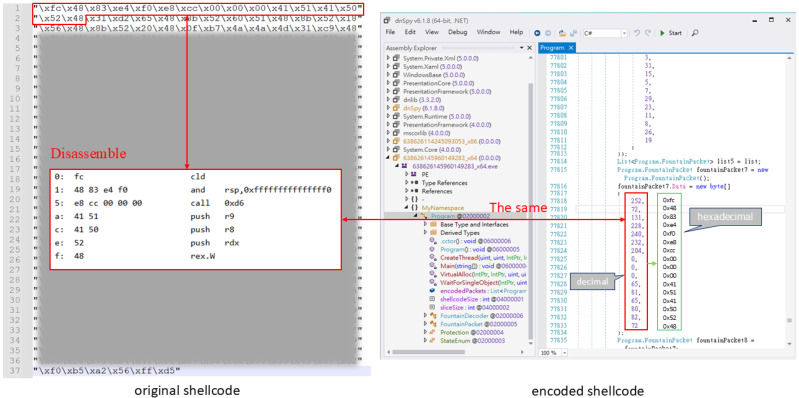
A comparison of 64-bit shellcode before and after encoding in the worst-case scenario.

Similarly, Figure 2 illustrates the comparison for the 64-bit shellcode. The first 14 bytes of the original and encoded shellcode remain unchanged (in decimal format), indicating that encoding scatters the data rather than encrypting it. This behavior, coupled with a larger slice size, generates a more extended shellcode structure, inadvertently providing antivirus software with additional features to classify the file as malicious.

**Figure 3:**
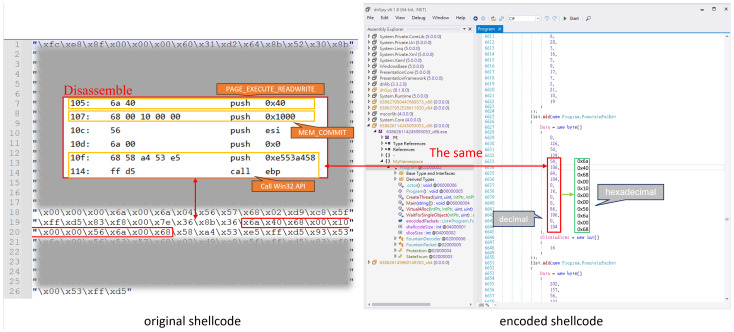
A comparison of 32-bit shellcode before and after encoding in the worst-case scenario.

**Figure 4:**
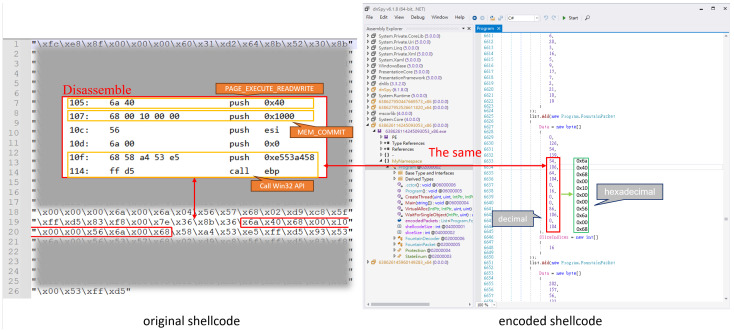
A comparison of 64-bit shellcode before and after encoding in the worst-case scenario.

In addition, the encoded shellcode does not sufficiently obfuscate key API call parameters, allowing security tools to identify it as malicious. As demonstrated in Figure 3 and Figure 4, the disassembled shellcode reveals clear patterns of API calls and their associated parameters. These patterns, when not sufficiently obscured, are strong indicators of malicious activity. For example, in both x86 and x64 shellcode, the parameters for memory allocation APIs such as VirtualAlloc can be directly identified. These parameters include the following:

- PAGE_EXECUTE_READWRITE (0x40), which grants executable permissions to allocated memory, a common feature of malicious payloads;
- MEM_COMMIT (0x1000), indicating the allocation type.

Additionally, the sequence of push, mov, and call instructions in the disassembly clearly prepares and invokes API calls, such as VirtualAlloc, through an indirect call ebp. This behavior is consistent with known malicious patterns in shellcode, where API addresses are resolved dynamically to evade static analysis.

Key Observations:

- **Parameter Consistency:** The hardcoded values for key API parameters (e.g., 0x40, 0x1000) are easily detectable and align with typical malicious shellcode behavior;
- **Indirect API Calls:** The use of indirect calls (e.g., call ebp) is a common obfuscation technique but can still be identified through dynamic analysis tools or advanced heuristic detection;
- **Entropy Disparity:** While the encoded shellcode increases overall entropy, certain regions, such as API call parameters, retain low entropy and remain detectable.

These findings highlight a critical flaw in the encoding process: it does not fully obfuscate key API call parameters, commonly used by antivirus engines to classify the payload as malicious.

### 5.2. Analysis of Encoding and Decoding Processes

Successful decoding relies on the following conditions:

1. The attacker must collect enough packets (at least *N* independent packets, where *N* is the number of slices);
2. The attacker must know the index set $I_{k}$ of each packet;
3. The attacker must know the initial seed value *s* used in the XOR operation.

Challenges for attackers:

1. **Packet Combination Space:** Given *M* packets, if M≥N, the number of possible combinations is
  (1) MN,
  which grows exponentially as M≫N. For example, 1000200≈10298.
2. **Seed Brute Force:** Since the seed *s* is a single byte, the attacker needs to try only $2^{8}=256$ possibilities.
3. **Overall Complexity:** Assuming each attempt involves solving *N* sparse linear equations, with a computational complexity of $O(N^{2})$, the total brute-force complexity is as follows:
  (2) Tbrute-force=MN·28·O(N2).

Analysis of low success rate:

The decoding process depends on whether the matrix *A* is full-rank. For randomly selected index sets $I_{k}$, the success probability of decoding is approximately

(3) $$P_{\mathrm{success}}=1-\prod_{k=1}^{N}{\left(1-\frac{k}{N}\right)}^{M}.$$

when M≈N, the success rate drops significantly.

Information-Theoretic Proofs:

- **Entropy Increase and Obfuscation:** After fountain code encoding, the entropy *H* of the packet data approaches the maximum entropy $H_{\max}$:
  (4) $$H_{\mathrm{norm}}=\frac{H}{H_{\max}},\;H_{\mathrm{norm}}\to 1.$$
  Attackers cannot extract meaningful patterns through statistical analysis.
- **Irreversibility of XOR:** Without knowledge of *s* and packet indices, attackers cannot reconstruct valid slices.

### 5.3. Future Studies

During testing, we tried to preserve Meterpreter’s characteristics by employing the presented strategy to eliminate distinguishing traits. Although this experiment focused on randomly encoding and creating unique samples, more strategies should be included in future studies. Encoding unique harmful features on network traffic [39] requires adjusting or developing our own shellcode to identify command and control (C2), even though this can be accomplished using fileless malware [40]. The anti-VM approach should also be considered [41]. It can integrate different methods, making forensic and reverse engineering more difficult [42]. This approach can also be used for PowerShell scripts [43] and to obtain packet data from the internet.

### 5.4. Limitations

Our study has specific limitations. One limitation pertains to the Microsoft Windows operating system, which requires the installation of the .NET Framework to run the virus. The .NET Framework is pre-installed on contemporary operating systems, but earlier ones require manual installation. This experiment may be executed using .NET Core using the C# programming language, and it is compatible with installation and execution on a Linux system [44]. The mmap function from libc.so.6 is analogous to the Win32 API VirtualAlloc, while Marshal.GetDelegateForFunctionPointer from System.Runtime.InteropServices is analogous to the Win32 API CreateThread.

## 6. Conclusions

This study utilizes fountain code to obfuscate shellcode, enhancing its resistance to detection by security systems. The proposed encoding method incorporates randomness, markedly enhancing entropy and diminishing detection rates among multiple antivirus systems and thus demonstrating its efficacy in evasion. The method streamlines decoding, rendering it appropriate for shellcode production pipelines; however, the encoding process is slower than that of more advanced techniques like LT or Raptor codes. The simplicity of fountain code facilitates its practicality and ease of integration, balancing usability and evasion capability. The results emphasize the importance of parameter tuning, especially in the optimization of the slice size, packet count, and entropy. Increased slice sizes and packet counts enhance randomness and decoding success, but they also lead to larger file sizes. This highlights the significance of selecting parameters strategically according to specific use cases, thus optimally balancing evasion effectiveness and resource efficiency.

## Figures and Tables

**Table 1:** The shellcode generated from Msfvenom.

Architecture	Msfvenom Command
x86	msfvenom -p /windows/meterpreter/reverse_tcp LHOST=<LHOST> LPORT=4444 -f c
x64	msfvenom -p /windows/x64/meterpreter/reverse_tcp LHOST=<LHOST> LPORT=4444 -f c

**Table 2:** Entropy results sorted by packet count and slice size for x86 Meterpreter reverse shell with lower value.

Packets	Slice Size	Success Decoding	Shellcode Entropy	Encode Entropy (Min)	Encode Entropy (Max)	File Size (Avg)
250	4	0	0.896235027	-	-	-
250	8	0	0.896235027	-	-	-
250	16	24	0.896235027	0.98503051	0.992427675	57 kb
250	32	25	0.896235027	0.973816785	0.987966061	53 kb
250	64	25	0.896235027	0.948876115	0.967126066	55 kb
500	4	0	0.896235027	-	-	-
500	8	18	0.896235027	0.989671667	0.993759997	127 kb
500	16	25	0.896235027	0.988779078	0.994360988	106 kb
500	32	25	0.896235027	0.978403054	0.988017714	101 kb
500	64	25	0.896235027	0.955413881	0.964298565	106 kb
750	4	0	0.896235027	-	-	-
750	8	25	0.896235027	0.991766781	0.995962125	185 kb
750	16	25	0.896235027	0.991622706	0.994991391	157 kb
750	32	25	0.896235027	0.979355781	0.986933525	149 kb
750	64	25	0.896235027	0.95449703	0.965643532	156 kb
1000	4	0	0.896235027	-	-	-
1000	8	25	0.896235027	0.993825658	0.996373339	245 kb
1000	16	25	0.896235027	0.99150072	0.995442883	206 kb
1000	32	25	0.896235027	0.981085998	0.986335313	197 kb
1000	64	25	0.896235027	0.956103917	0.965333322	207 kb

**Table 3:** Entropy results sorted by packet count and slice size for x64 Meterpreter reverse shell with lower value.

Packets	Slice Size	Success Decoding	Shellcode Entropy	Encode Entropy (Min)	Encode Entropy (Max)	File Size (Avg)
250	4	0	0.866219586	-	-	-
250	8	0	0.866219586	-	-	-
250	16	17	0.866219586	0.989701663	0.994201156	61 kb
250	32	25	0.866219586	0.987495503	0.992933672	55 kb
250	64	25	0.866219586	0.973681544	0.984492352	57 kb
500	4	0	0.866219586	-	-	-
500	8	0	0.866219586	-	-	-
500	16	25	0.866219586	0.993458624	0.996361258	116 kb
500	32	25	0.866219586	0.991145936	0.99484566	105 kb
500	64	25	0.866219586	0.97648581	0.982825214	110 kb
750	4	0	0.866219586	-	-	-
750	8	3	0.866219586	0.995092963	0.995291433	210 kb
750	16	25	0.866219586	0.994864082	0.996855637	170 kb
750	32	25	0.866219586	0.990616701	0.995384983	157 kb
750	64	25	0.866219586	0.976686746	0.98352951	162KB
1000	4	0	0.866219586	-	-	-
1000	8	19	0.866219586	0.995025236	0.996902249	283 kb
1000	16	25	0.866219586	0.995511946	0.997440456	226 kb
1000	32	25	0.866219586	0.992070581	0.995239605	208 kb
1000	64	25	0.866219586	0.978209475	0.983397851	215 kb

**Table 4:** Entropy results sorted by packet count and slice size for x86 Meterpreter reverse shell with higher value.

Packets	Slice Size	Success Decoding	Shellcode Entropy	Encode Entropy (Min)	Encode Entropy (Max)	File Size (Avg)
1000	8	25	0.896235027	0.994232367	0.996849737	317 kb
1000	16	25	0.896235027	0.992861211	0.995433089	270 kb
1000	32	25	0.896235027	0.986183107	0.990486731	225 kb
1000	64	25	0.896235027	0.961186269	0.967656705	118 kb
1000	128	25	0.896235027	0.93369074	0.942361528	65 kb
2000	8	25	0.896235027	0.995794869	0.997679587	620 kb
2000	16	25	0.896235027	0.993985397	0.996148508	528 kb
2000	32	25	0.896235027	0.98625613	0.989079059	420 kb
2000	64	25	0.896235027	0.961123457	0.967412767	206 kb
2000	128	25	0.896235027	0.935762935	0.941693808	121 kb
3000	8	25	0.896235027	0.996754133	0.9980809	923 kb
3000	16	25	0.896235027	0.994238917	0.996299434	780 kb
3000	32	25	0.896235027	0.987049086	0.98951869	609 kb
3000	64	25	0.896235027	0.962440835	0.966613312	290 kb
3000	128	25	0.896235027	0.937016187	0.942564261	177 kb
4000	8	25	0.896235027	0.997256016	0.998076397	1226 kb
4000	16	25	0.896235027	0.994823349	0.996568556	1031 kb
4000	32	25	0.896235027	0.98675825	0.989284019	790 kb
4000	64	25	0.896235027	0.962201556	0.965914419	363 kb
4000	128	25	0.896235027	0.936493392	0.940475369	233 kb

**Table 5:** Entropy results sorted by packet count and slice size for x64 Meterpreter reverse shell with higher value.

Packets	Slice Size	Success Decoding	Shellcode Entropy	Encode Entropy (Min)	Encode Entropy (Max)	File Size (Avg)
1000	8	20	0.866219586	0.995523813	0.997074305	356 kb
1000	16	25	0.866219586	0.995539811	0.997245624	293 kb
1000	32	25	0.866219586	0.993494934	0.996862112	256 kb
1000	64	25	0.866219586	0.983122616	0.987486378	164 kb
1000	128	25	0.866219586	0.952280556	0.959454628	71 kb
2000	8	25	0.866219586	0.997178983	0.99824582	700 kb
2000	16	25	0.866219586	0.996711798	0.99796297	575 kb
2000	32	25	0.866219586	0.993356884	0.995728772	492 kb
2000	64	25	0.866219586	0.982385729	0.985924599	298 kb
2000	128	25	0.866219586	0.95374076	0.959051457	128 kb
3000	8	25	0.866219586	0.997754239	0.998564097	1041 kb
3000	16	25	0.866219586	0.996905775	0.998097463	852 kb
3000	32	25	0.866219586	0.994283511	0.995903518	728 kb
3000	64	25	0.866219586	0.983241022	0.986679814	412 kb
3000	128	25	0.866219586	0.954248628	0.958669975	187 kb
4000	8	25	0.866219586	0.997948703	0.998805126	1387 kb
4000	16	25	0.866219586	0.997458472	0.998204189	1140 kb
4000	32	25	0.866219586	0.994572898	0.99583737	950 kb
4000	64	25	0.866219586	0.983957472	0.985740059	530 kb
4000	128	25	0.866219586	0.955824921	0.958622307	244 kb

**Table 6:** Virus scan results for original and encoded shellcode.

Type	Detected	Detail	Size
Original Reverse Shell (x86)	18/39	Meterpreter detected it.	6 kb
Original Reverse Shell (x64)	16/39	Meterpreter detected it.	5 kb
Best Case for Encoding Reverse Shell (x86)	3/39	Only 3 vendors detected.	270 kb
Best Case for Encoding Reverse Shell (x64)	1/39	Only 1 vendor detected.	293 kb
Worst Case for Encoding Reverse Shell (x86)	12/39	Despite no Meterpreter description, the file was malicious.	56 kb
Worst Case for Encoding Reverse Shell (x64)	5/39	Despite no Meterpreter description, the file was malicious.	456 kb

**Table 7:** Antivirus scan results for original and encoded (x86) shellcode in the best-case scenario.

No.	AntiVirus	Original (x86 Shellcode)	Encoded (x86 Shellcode)
1	AdAware	Generic.ShellCode.Marte.3.47C3E1F2	-
2	Arcabit	Generic.ShellCode.Marte.3.47C3E1F2	-
3	Avast	Win32:Meterpreter-C	-
4	AVG	Win32:Meterpreter-C	-
5	Avira	HEUR/AGEN.1234830	HEUR/AGEN.1234954
6	ClamAV	Win.Malware.Swrort-9872015-0	-
7	Emsisoft	Generic.ShellCode.Marte.3.47C3E1F2	-
8	F-Secure	-	Heuristic.HEUR/AGEN.1305448
9	G Data	Generic.ShellCode.Marte.3.47C3E1F2	-
10	IKARUS	Trojan.Win64.Rozena	-
11	Immunet	Win.Malware.Swrort-9872015-0	-
12	Microsoft Defender	Trojan:Win32/Meterpreter.RPZ!MTB	-
13	NOD32	MSIL/Rozena.FW trojan	MSIL/Rozena.T trojan
14	Norman	Win32:Meterpreter-C	-
15	Sophos	Mal/Swrort-W	-
16	VirITExplorer	Trojan.Win32.Rozena.AA	-
17	VirusFighter	ATK/Swrort-W	-
18	ZoneAlarm	HEUR:Trojan.Win32.Generic	-

**Table 8:** Antivirus scan results for original and encoded (x64) shellcode in the best-case scenario.

No.	AntiVirus	Original (x64 Shellcode)	Encoded (x64 Shellcode)
1	AdAware	Generic.ShellCode.Marte.4.F9919BE1	-
2	Arcabit	Generic.ShellCode.Marte.4.F9919BE1	-
3	Avast	Win32:MsfShell-V	-
4	AVG	Win32:MsfShell-V	-
5	Avira	HEUR/AGEN.1234848	-
6	ClanAV	Win.Malware.Metasploit-10022275-0	-
7	Emsisoft	Generic.ShellCode.Marte.4.F9919BE1	-
9	G Data	Generic.ShellCode.Marte.4.F9919BE1	-
10	IKARUS	Trojan.Win64.Meterpreter	-
A	Immunet	Win.Malware.Metasploit-10022275-0	-
11	Microsoft Defender	Trojan:Win64/Meterpreter.B	-
12	NOD32	Win64/Rozena.JN trojan	MSIL/Rozena.FW.gen trojan
13	Norman	Win32:MsfShell-V	-
14	VirITExplorer	Trojan.Win64.MSIL_Heur.A	-
15	VirusFighter	ATK/Swrort-W	-
16	ZoneAlarm	HEUR:Trojan.Win32.Generic	-

**Table 9:** Antivirus detection results for original and encoded x86 shellcode in the worst-case scenario.

No.	AntiVirus	Original (x86 Shellcode)	Encoded (x86 Shellcode)
1	AdAware	Generic.ShellCode.Marte.3.AFB20B04	Gen:Variant.Razy.461207
2	Alyac	-	Gen:Variant.Razy.461207
3	Arcabit	Generic.ShellCode.Marte.3.AFB20B04	Trojan.Razy.D70997
4	Avast	Win32:Meterpreter-C	-
5	AVG	Win32:Meterpreter-C	-
6	Avira	TR/Rozena.Gen	HEUR/AGEN.1234955
7	Bullguard	Win32:Meterpreter-C	-
8	Crowdstrike Falcon	Threat detected	Threat detected
9	Emsisoft	-	Gen:Variant.Razy.461207
10	F-Secure	Trojan.TR/Rozena.Gen	Heuristic.HEUR/AGEN.1305448
11	G Data	Generic.ShellCode.Marte.3.AFB20B04	Gen:Variant.Razy.461207
12	IKARUS	Trojan.Win64.Rozena	-
13	Microsoft Defender	Trojan:Win32/Meterpreter.RPZ!MTB	Trojan:MSIL/Rozena.HNF!MTB
14	NOD32	MSIL/Rozena.FW trojan	MSIL/Rozena.T trojan
15	Norman	Win32:Meterpreter-C	-
16	SecureAge APEX	Unknown	-
17	Sophos	Mal/Swrort-W	Mal/MSIL-KC
18	VirITExplorer	Trojan.Win32.Rozena.AA	-
19	VirusFighter	ATK/Swrort-W	Mal/MSIL-KC
20	ZoneAlarm	HEUR:Trojan.Win32.Generic	-

**Table 10:** Antivirus detection results for original and encoded x64 shellcode in the worst-case scenario.

No.	AntiVirus	Original (x64 Shellcode)	Encoded (x64 Shellcode)
1	AdAware	Generic.ShellCode.Marte.4.BD082403	-
2	Arcabit	Generic.ShellCode.Marte.4.BD082403	-
3	Avast	Win32:MsfShell-V	-
4	AVG	Win32:MsfShell-V	-
5	Avira	TR/Rozena.Gen	HEUR/AGEN.1252172
6	Bullguard	Win32:MsfShell-V	-
7	Crowdstrike Falcon	Threat detected	Threat detected
8	F-Secure	Trojan.TR/Rozena.Gen	-
9	G Data	Generic.ShellCode.Marte.4.BD082403	-
10	IKARUS	Trojan.Win64.Rozena	-
11	Microsoft Defender	Trojan:Win64/Meterpreter.B	Trojan:MSIL/Rozena.HNF!MTB
12	NOD32	Win64/Rozena.JN trojan	MSIL/Rozena.FW.gen trojan
13	Norman	Win32:MsfShell-V	-
14	SecureAge APEX	Malicious	-
15	VirusFighter	ATK/Swrort-W	-
16	ZoneAlarm	HEUR:Trojan.Win32.Generic	HEUR:Trojan.Win32.Generic

## Data Availability

Data are contained within the article.

## Appendix A. Details

### Appendix A.1. Scan Details for Original and Evasion Tools

**Table A1:** Antivirus scan results for original and evasion (x86) tools.

No.	AntiVirus	Msfvenom Staged Reverse Shell (*.exe)	shikata_ga_nai with 10 Iterators	shikata_ga_nai with 20 Iterators	Veil-Evasion in C#
1	AdAware	Trojan.CryptZ.Marte.1.Gen	Trojan.CryptZ.Marte.1.Gen	Trojan.CryptZ.Marte.1.Gen	Gen:Variant.Kryptik.175
2	Alyac	Trojan.CryptZ.Gen	Trojan.CryptZ.Gen	Trojan.CryptZ.Gen	Gen:Variant.Razy.575638
3	Arcabit	Trojan.CryptZ.Marte.1.Gen	Trojan.CryptZ.Marte.1.Gen	Trojan.CryptZ.Marte.1.Gen	Trojan.Kryptik.175
4	Avast	Win32:Meterpreter-C	Win32:SwPatch	Win32:ShikataGaNai-C	Win32:Evo-gen
5	AVG	Win32:Meterpreter-C	Win32:SwPatch	Win32:ShikataGaNai-C	Win32:Evo-gen
6	Avira	TR/Patched.Gen2	TR/Patched.Gen2	TR/Patched.Gen2	TR/Crypt.XPACK.Gen7
7	CalmAV	Win.Trojan.Swrort-5710536-0	Win.Trojan.MSShellcode-6360728-0	Win.Trojan.MSShellcode-6360728-0	-
8	Comodo Linux	TrojWare.Win32.Rozena.A	TrojWare.Win32.Rozena.A	TrojWare.Win32.Rozena.A	TrojWare.MSIL.TrojanDownloader.Small.H
9	Emsisoft	Trojan.CryptZ.Marte.1.Gen	Trojan.CryptZ.Marte.1.Gen	Trojan.CryptZ.Marte.1.Gen	Gen:Variant.Kryptik.175
10	F-Prot	W32/Swrort.A.gen!Eldorado	W32/Swrort.A.gen!Eldorado	W32/Swrort.A.gen!Eldorado	-
11	F-Secure	Trojan.TR/Patched.Gen2	Trojan.TR/Patched.Gen2	Trojan.TR/Patched.Gen2	Trojan.TR/Crypt.XPACK.Gen7
12	G Data	Trojan.CryptZ.Marte.1.Gen	Trojan.CryptZ.Marte.1.Gen	Trojan.CryptZ.Marte.1.Gen	Gen:Variant.Kryptik.175
13	IKARUS	Trojan.Win32.Swrort	Trojan.Win32.Swrort	Trojan.Win32.Swrort	Trojan-Downloader.MSIL.Tiny
14	Immunet	Win.Trojan.Swrort-5710536-0	Win.Trojan.MSShellcode-6360728-0	Win.Trojan.MSShellcode-6360728-0	-
15	Max Secure	Packed_Win32_BDF_a_1_YR	Packed_Win32_BDF_a_1_YR	Packed_Win32_BDF_a_1_YR	-
16	McAfee	Swrort.i trojan !!!	Swrort.i trojan !!!	Swrort.i trojan !!!	Trojan-Veil-FLRH!A132AFFEC851 trojan !!!
17	Microsoft Defender	Trojan:Win32/Meterpreter.RPZ!MTB	Trojan:Win32/Meterpreter.A	Trojan:Win32/Meterpreter.A	Trojan:MSIL/Tiny.EM!MTB
18	NANO	Virus.Win32.Gen-Crypt.ccnc	Virus.Win32.Gen.ccmw	Virus.Win32.Gen-Crypt.ccnc	Multiple Threats Detected
19	NOD32	Win32/Rozena.AA trojan	Win32/Rozena.AA trojan	Win32/Rozena.AA trojan	MSIL/TrojanDownloader.Tiny.BQ trojan
20	Norman	Win32:Meterpreter-C	Win32:SwPatch	Win32:ShikataGaNai-C	Win32:Evo-gen
21	Quick Heal	Trojan.Swrort.A	Trojan.Swrort.A	Trojan.Swrort.A	-
22	SecureAge APEX	Malicious	Malicious	Malicious	Malicious
23	Seqrite	Trojan.Swrort.A	Trojan.Swrort.A	Trojan.Swrort.A	-
24	Sophos	Mal/EncPk-TZ	Mal/EncPk-TZ	Mal/EncPk-TZ	Troj/Rozena-D
25	TrendMicro	-	BKDR_SWRORT.SM	BKDR_SWRORT.SM	-
26	VirITExplorer	Trojan.Win32.Rozena.AA	Trojan.Win32.Rozena.AA	Trojan.Win32.Rozena.AA	-
27	VirusFighter	Mal/EncPk-ACE	Mal/EncPk-ACE	Mal/EncPk-ACE	ATK/Dloadr-EFR
28	Zillya	Trojan.RozenaGen.Win32.2	Trojan.RozenaGen.Win32.2	Trojan.RozenaGen.Win32.2	-
29	ZoneAlarm	HEUR:Trojan.Win32.Generic	HEUR:Trojan.Win32.Generic	HEUR:Trojan.Win32.Generic	HEUR:Trojan.Win32.Generic

**Table A2:** Antivirus scan results for original and evasion (x64) tools.

No.	AntiVirus	Msfvenom Staged Reverse Shell (*.exe)	shikata_ga_nai with 10 Iterators	shikata_ga_nai with 20 Iterators	PwnWind Module in TheFatRat
1	AdAware	Trojan.Metasploit.A	Trojan.Metasploit.A	Trojan.Metasploit.A	Gen:Variant.Zusy.486491
2	Alyac	Trojan.Metasploit.A	Trojan.Metasploit.A	Trojan.Metasploit.A	-
3	Arcabit	Trojan.Metasploit.A	Trojan.Metasploit.A	Trojan.Metasploit.A	Trojan.Zusy.D76C5B
4	Avast	Win32:MsfShell-V	Win64:Evo-gen	Win64:Evo-gen	Win64:TrojanX-gen
5	AVG	Win32:MsfShell-V	Win64:Evo-gen	Win64:Evo-gen	Win64:TrojanX-gen
6	Avira	TR/Crypt.XPACK.Gen7	TR/Crypt.XPACK.Gen7	TR/Crypt.XPACK.Gen7	-
7	CalmAV	Win.Malware.Metasploit-10022275-0	Win.Trojan.MSShellcode-6360728-0	Win.Trojan.MSShellcode-6360730-0	Win.Malware.Agent-9870952-0
8	Crowdstrike Falcon	-	Threat detected.	-	-
8	Emsisoft	Trojan.Metasploit.A	Trojan.Metasploit.A	Trojan.Metasploit.A	Gen:Variant.Zusy.486491
9	F-Prot	W64/S-c4a4ef26!Eldorado	W64/S-c4a4ef26!Eldorado	W64/S-c4a4ef26!Eldorado	-
10	F-Secure	Trojan.TR/Crypt.XPACK.Gen7	Trojan.TR/Crypt.XPACK.Gen7	Trojan.TR/Crypt.XPACK.Gen7	-
11	G Data	Trojan.Metasploit.A	Trojan.Metasploit.A	Trojan.Metasploit.A	Gen:Variant.Zusy.486491
12	IKARUS	Trojan.Win64.Meterpreter	Trojan.Win64.Meterpreter	Trojan.Win64.Meterpreter	-
13	Immunet	Win.Malware.Metasploit-10022275-0	Win.Trojan.MSShellcode-6360728-0	Win.Trojan.MSShellcode-6360730-0	Win.Malware.Agent-9870952-0
14	Kaspersky	Trojan.Win64.Packed.gen	Trojan.Win64.Packed.gen	Trojan.Win64.Packed.gen	-
15	Max Secure	Trojan_W64_090622_Packed_gen_YR	Trojan_W64_090622_Packed_gen_YR	Trojan_W64_090622_Packed_gen_YR	-
16	McAfee	Trojan-FJIN!102811008CF1 trojan !!!	Trojan-FJIN!F999F6FE19FA trojan !!!	Trojan-FJIN!765AE5EF0526 trojan !!!	-
17	Microsoft Defender	Trojan:Win64/Meterpreter!pz	Trojan:Win64/Meterpreter!pz	Trojan:Win64/Meterpreter!pz	Trojan:Win64/Rozena.IG!MTB
18	NOD32	Win64/Rozena.M trojan	Win64/Rozena.J trojan	Win64/Rozena.J trojan	Win64/Rozena.HH trojan
19	Norman	Win32:MsfShell-V	Win64:Evo-gen	Win64:Evo-gen	Win64:TrojanX-gen
20	Quick Heal	HackTool.Metasploit.S9212471	HackTool.Metasploit.S9212471	HackTool.Metasploit.S9212471	-
21	SecureAge APEX	Malicious	Malicious	Malicious	-
22	Seqrite	Trojan.Dynamer.S4605	Trojan.Dynamer.S4605	Trojan.Dynamer.S4605	-
23	Sophos	Mal/Swrort-J	Mal/Swrort-J	Mal/Swrort-J	-
24	VirITExplorer	Trojan.Win32.Generic.BZPS	Trojan.Win32.Generic.BZPS	Trojan.Win32.Generic.BZPS	-
25	VirusFighter	ATK/Meter-A	ATK/Swrort-J	ATK/Swrort-J	ATK/FatRat-G
26	Zillya	-	-	-	Trojan.Rozena.Win64.43244
27	ZoneAlarm	HEUR:Trojan.Win64.Packed.gen	HEUR:Trojan.Win64.Packed.gen	HEUR:Trojan.Win64.Packed.gen	HEUR:Trojan.Win32.Generic

### Appendix A.2. Scan Report from Kleenscan.com

**Table A3:** The scan report for this experiment.

Notes	Scan Result URL (all of the URLs were accessed on 22 October 2024)
Msfvenom x86 staged reverse shell (*.exe)	https://kleenscan.com/scan_result/122248ba215ef30f18b8add2c73a3438f509b27b601173f0e49fe59df6eb6830
Msfvenom x86 staged reverse shell (*.exe) with shikata_ga_nai with 10 iterators	https://kleenscan.com/scan_result/64b9bc4731e4bddc84f32f92e56f1fff96f846dae282a5adede8075548fa84d7
Msfvenom x86 staged reverse shell (*.exe) with shikata_ga_nai with 20 iterators	https://kleenscan.com/scan_result/deb71523ea206bc4ca1238e3292fbc4db0fe7cbe47bcf7865637d95fa3e70e54
Veil-evasion in C#	https://kleenscan.com/scan_result/d9278171ce04185fefb9f867ea2a40f0c65b182d62aa7a1ae00b4a2ba8c19001
Msfvenom x64 staged reverse shell (*.exe)	https://kleenscan.com/scan_result/92d96c81788df01ef0b9fe3b253b12f00f3893f0014c86672b56a59546f89627
Msfvenom x64 staged reverse shell (*.exe) with shikata_ga_nai with 10 iterators	https://kleenscan.com/scan_result/8e5e2c26e6fdd4f164d42250314a40cbf99f1c98c142006d0c924e87a8cc5aa7
Msfvenom x64 staged reverse shell (*.exe) with shikata_ga_nai with 20 iterators	https://kleenscan.com/scan_result/f08f1c4227818a7e7614ee58eff0e8c1eb17415a77d0bb8ec9bf8a62929b001d
PwnWind module in TheFatRat	https://kleenscan.com/scan_result/747d181b5e3c8a40954186118a794c13ccf5f010edc3876bd8c18ec5e8a4589e
Original (x86 shellcode)	https://kleenscan.com/scan_result/e4ed251d8d60be17cda1e62fe0cab9d03dc99cb5d56c22fd0652422c4dcb74aa
Encoded shellcode in best case (x86 shellcode)	https://kleenscan.com/scan_result/fb5d4c6dd5261174ce9d0cd055cfa77e17ca4a96c84d936df18a64cfd1bf4c37
Original (x64 shellcode)	https://kleenscan.com/scan_result/bb9067923d300c3e02efa7c5279e5d0eb80a6d2b7145119a0947c45ad8a01d51
Encoded shellcode in best case (x64 shellcode)	https://kleenscan.com/scan_result/c396a8f72a94023fd4d00287a55b04c39ad9f1ee9ea069c8462c647329d3eb6b
Encoded shellcode in worst case (x86 shellcode)	https://kleenscan.com/scan_result/580d89a95703d090adb2bfd3217f4859d646a8b18a0a1e442c0e6d85bbe73f4c
Encoded shellcode in worst case (x64 shellcode)	https://kleenscan.com/scan_result/6e6ddde9f0f0a385aff4ccf007a8cbb76e16550a3dbf470cd0baef9f0baf7746

### Appendix A.3. The Implemented Pseudocode

**Listing 1.** Pseudocode for executing the decoded shellcode using FountainDecoder.
Function ExecuteShellcode() // *Initialize FountainDecoder* Set decoder = Initialize FountainDecoder WITH: - shellcodeSize - sliceSize -~seed // *Receive encoded packets* FOR EACH packet IN encodedPackets Call ReceivePacket(packet) END~FOR // *Decode packets to reconstruct shellcode* Set shellcode = Call Decode() // *Allocate memory for shellcode* Set funcAddr = Call VirtualAlloc WITH: - BaseAddress: NULL - Size: LENGTH(shellcode) - State: MEM_COMMIT - Protection: PAGE_EXECUTE_READWRITE // *Copy decoded shellcode to allocated memory* Call Marshal.Copy WITH: - Source: shellcode - Destination: funcAddr - Length: LENGTH(shellcode) // *Initialize thread parameters* Set hThread = NULL Set threadId = 0 Set pinfo = NULL // *Create a thread to execute shellcode* Set hThread = Call CreateThread WITH: - StartAddress: funcAddr - Parameter: pinfo - Flags: 0 - ThreadId: threadId // *Wait for the thread to complete* Call WaitForSingleObject WITH: - Handle: hThread - Timeout: INFINITE Return End Function
